# Beneficial Effects of Early Time-Restricted Feeding on Metabolic Diseases: Importance of Aligning Food Habits with the Circadian Clock

**DOI:** 10.3390/nu13051405

**Published:** 2021-04-22

**Authors:** Anouk Charlot, Fanny Hutt, Eugénie Sabatier, Joffrey Zoll

**Affiliations:** CRBS, UR3072: Mitochondria, Oxidative Stress and Muscular Protection, University of Strasbourg, 1 rue Eugène Boeckel, 67000 Strasbourg, France; fanny-04@live.fr (F.H.); eugenie.sabatier@etu.unistra.fr (E.S.)

**Keywords:** time-restricted fasting, intermittent fasting, circadian clock, metabolic diseases, obesity, cardiovascular disease

## Abstract

The importance of metabolic health is a major societal concern due to the increasing prevalence of metabolic diseases such as obesity, diabetes, and various cardiovascular diseases. The circadian clock is clearly implicated in the development of these metabolic diseases. Indeed, it regulates physiological processes by hormone modulation, thus helping the body to perform them at the ideal time of day. Since the industrial revolution, the actions and rhythms of everyday life have been modified and are characterized by changes in sleep pattern, work schedules, and eating habits. These modifications have in turn lead to night shift, social jetlag, late-night eating, and meal skipping, a group of customs that causes circadian rhythm disruption and leads to an increase in metabolic risks. Intermittent fasting, especially the time-restricted eating, proposes a solution: restraining the feeding window from 6 to 10 h per day to match it with the circadian clock. This approach seems to improve metabolic health markers and could be a therapeutic solution to fight against metabolic diseases. This review summarizes the importance of matching life habits with circadian rhythms for metabolic health and assesses the advantages and limits of the application of time-restricted fasting with the objective of treating and preventing metabolic diseases.

## 1. Introduction

Obesity is a growing and epidemic disease, with a prevalence reaching such proportions that it is now considered as a pandemic. Obese people expose themselves to health problems. Indeed, they are more susceptible to suffering from heart attacks, strokes, and diabetes [1]. Nowadays, there is no miracle cure to treat obesity. The few pharmacological agents available usually present side effects and need to be combined with a dietary intervention, an increase of physical activity, and behavior modifications [2,3]. When lifestyle and pharmacotherapy approaches result in insufficient weight loss or insufficient maintenance of weight loss, surgery may be proposed to patients with morbid obesity or severe obesity associated with comorbidities. Bariatric surgery promotes weight loss by reducing stomach volume to limit nutritional intake. This gastric reduction can also be combined with a malabsorptive procedure where a part of the intestine is resected [4]. Although bariatric surgery is an effective therapeutic option for obese patients and is used with increasing frequency, this procedure is associated with serious complications, such as gastroesophageal reflux disease, hypoglycemia, osteoporosis, and nutritional deficiencies [5].

Public health policies, scientific recommendations, and the guidance of health organizations also try to fight against obesity with preventative care approaches [6]. For example, nutrition labeling is used on food packages to inform consumers, providing them with simplified information on the essential nutrients contained in the food products. It helps them to identify and promote healthy eating by choosing healthy foods [7]. Informational campaigns, such as “five fruits and vegetables per day” or graphic images that warn about the consequences of a poor diet, are broadcasted across a variety of media platforms to promote healthy eating behaviors [8]. It is now inscribed in the social conscious that food quality and dietary habits are essential to a healthy lifestyle. However, “what we eat” is not everything; “when we eat” also has a significant role in metabolic health. The circadian rhythms, which regulate the organism’s functions, also determine at what time of day it is most appropriate to eat.

In this review, we discuss the importance of the circadian clock in physiologic processes, and how disruptions of it can lead to metabolic impairments. We also focus on the interest of using intermittent fasting to “synchronize” lifestyle habits with the circadian clock and suggest it as a therapeutic approach to fight against obesity and its associated complications.

## 2. Obesity and Diabetes Mellitus: Epidemiology and Physiopathology

In the past few decades, obesity prevalence has considerably increased: in 1975, 5% of the world population was obese, compared to 13% in 2016, according to the WHO database [1]. Obesity is defined as an excessive fat accumulation that leads to health complications [1].

The development of obesity is exacerbated by a modern diet rich in sugar and lipids. This type of hypercaloric food promotes hyperglycemia and insulin secretion that induce fat accumulation in adipose tissue [9].

Adipose tissue, in addition to its storage function, is an important endocrine organ that releases free fatty acids (FFA) which are partly responsible for insulin resistance [10]. FFA induces the inactivation of kinase proteins involved in the insulin signaling pathway by phosphorylation and leads to diabetes mellitus [11]. Adipose tissue produces plasminogen activator inhibitor type 1 (PAI-1), a protein involved in cardiovascular disease (CVD) development, particularly in the pathogenesis of atherothrombosis. Plasminogen allows for fibrinolysis, the mechanism that thins the blood. In the presence of PAI-1, plasminogen is inhibited and the fibrinolytic activity decreases, which increases the risk of thrombosis [12].

Although type 2 diabetes has been accurately described as a disease of insulin resistance, a large number of treatment centers around giving the patient more insulin. For instance, drugs all work by either increasing the endogenous production of insulin or increasing the amount of exogenous insulin received. While this works to reduce hyperglycemia in these patients, the concept of treating an insulin resistance disease by increasing insulin levels may be counterproductive, as it may later necessitate an increase in the dosage of medication over a long period of time (for review, please see: [13]). It appears that it is essential to find effective therapeutic solutions to treat obesity and diabetes and to reduce the spread of this pandemic of the 21st century. Dietary approaches, which are becoming increasingly popular, seem to be promising remedies to cure obesity and its comorbidities. Some new strategies that focus on the timing of eating and duration of fasting, rather than the type, quality, or quantity of foods, have been demonstrated to improve metabolic health independent of weight loss [14,15]. Indeed, a systematic review and meta-analysis concluded that intermittent fasting may provide a significant metabolic benefit by improving glycemic control, insulin resistance, and adipokine concentration as well as a reduction in the body mass index in adults [16].

## 3. Intermittent Fasting and Time-Restricted Feeding: Definition, History, and Principle

Intermittent fasting refers to the idea of adopting alternating fasting periods, characterized by a lack of food consumption, and feeding periods, during which food may be consumed ad libitum. Different forms of fasting are ingrained as normal behavior in animals to adapt to their ecologic and physiologic constraints, such as hibernation, molting, reproduction, illness, or intermittent feeding habits [17]. Voluntary abstinence from food has also been used throughout human history, especially in religious contexts such as the holy fasting observed by many during Ramadan or Lent [18]. It was also used as an epilepsy treatment in Ancient Rome, where the first mention of diet therapy to cure seizures dates back to 500 BC [19]. Currently, intermittent fasting is becoming increasingly popular because it seems to be an interesting clinical approach in the treatment of several diseases such as diabetes, obesity, cancer, or neurodegeneration (for review, please see: [20,21]). Many studies have shown the positive effects of intermittent fasting practice in the management of obesity and diabetes, principally by improving weight loss and metabolic markers such as the level of triglycerides, cholesterol, and glycemia, (for meta-analysis, please see: [22,23,24]). Nonetheless, several other authors did not observe any significant effects of intermittent fasting on metabolic health [25,26,27]. The evaluation of fasting efficacy during a year on obese adults also did not show improvement in risk indicators for cardiovascular disease [28]. Other studies have shown short-term effects of intermittent fasting because the weight lost during the fasting period was quickly regained after it [29,30].

If the effects of intermittent fasting are heterogenous, it is partly because the term “intermittent fasting” encompasses several approaches to the regulation of eating patterns which are not all equally effective. Some of these methods use alternation between a fasting day and feeding day (others split weeks into five days of normal dieting and two days of caloric restriction, known as “the 5:2 diet”, or two days of 24-h fasting, named “eat-stop-eat”). Generally, intermittent fasting restricts the feeding window from 6 to 10 h each day. Again, not all studies show the same positive results [31]. One of the reasons that could explain the controversial results of the fasting practices is that these methods do not consider the importance of matching the food intake timing with the circadian clock.

Indeed, even if fasting may appear to be a voluntary practice, linked to beliefs, culture, or environmental restriction, it is actually a biological process present in all living organisms, from archaea to mammals. Organisms are subject to circadian rhythms (CR—approximately 24-h oscillations) which are useful in facilitating the performance of physiological processes at the optimal times of day. The daily rhythms of sleeping and activity depend on a complex interaction between endogenous cell autonomous molecular oscillators and exogenous factors such as daily exposure to light/darkness and feeding/fasting patterns [32,33]. This circadian clock is essential for health and provides the rhythms of function for many organ systems, such as the digestive, cardiovascular, endocrine, and reproductive systems [32]. Growing evidence shows that circadian clock disruption is the cause of many metabolic diseases such as obesity, diabetes, or CVD [34]. A solution could be the use of “time-restricted feeding”, which is based on a routine day pattern, with 14 to 18 h of fasting per day and a restriction of the daily eating window of 10 to 6 h. Indeed, this approach seems to present health benefits, when it aims to align daily food consumption with the circadian clock [24,35].

## 4. Circadian Clock, a Key Regulator of the Physiological Processes

Circadian systems are composed of a biological clock network, made of a central clock and peripherical clocks. The central clock is the hypothalamic suprachiasmatic nucleus (SCN) and acts as a master pacemaker of circadian rhythm production and maintenance throughout the body [36]. The most powerful regulator of the circadian rhythms is light. The retina detects photonic inputs and transduces them to the SCN which allows for the synchronization of tissue activities and behaviors with the day/night cycles. These circadian oscillations are generated by proteins encoded by a set of genes (Clock, Bmal1, Per and Cry, for example) which constitute a transcriptional-translational feedback loop (for review, please see: [37,38]). Even if the SCN is the master regulator of the circadian system, peripheral clocks (such as the liver, adipocyte, or pancreas clocks) can be uncoupled from SCN control by other exogenous components, such as food intake. It appears that alterations in normal feeding rhythm can affect the circadian system and induce metabolic disorders [39,40].

**Figure 1:**
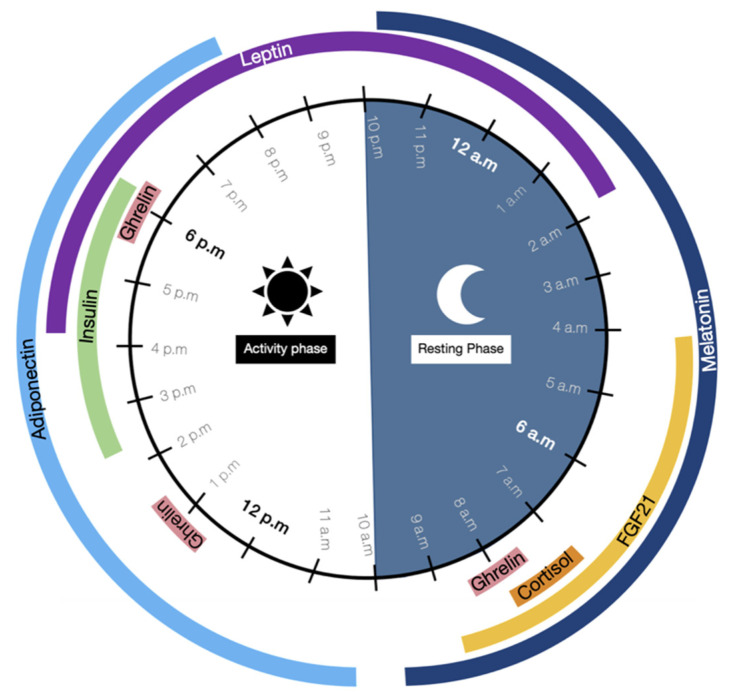
Time-of-day-dependent patterns of circulating hormone levels variation. Circadian rhythms are driven by oscillations of endocrine factor levels during the day. These variations are important to perform physiological processes at the optimal time of the day. Melatonin induces the resting phase whereas cortisol prepares the body for the activity phase. The other hormones drive modifications of glucose and lipid metabolism, promoting either catabolism with fatty acid oxidation and glycolysis (FGF21, adiponectin, leptin) or anabolism with lipogenesis and glycogenesis (insulin).

Circadian clocks play an essential role in glucose and lipid metabolism because they induce variations in circulating hormone levels, according to several stimuli (Figure 1). The production of hormones such as melatonin or cortisol depends on the SCN rhythmic activity in response to light/darkness whereas some others, known as nutrient-sensitive hormones, oscillate following a circadian basis but are also regulated by feeding/fasting cycles [41].

In human physiology, a day is split between an activity phase that begins at 10 a.m. and ends at 10 p.m., when the resting phase starts [32]. These two phases depend on melatonin, the “sleeping hormone”, renowned for its central role in the regulation of circadian rhythms. Melatonin oscillations have a particular characteristic pattern commonly used to define daily cycles. This hormone secretion begins approximately at 10 p.m., rises to its peak at 3 a.m., and finally decreases to its offset at 10 a.m. [42]. Melatonin secretion depends on the photoperiod and can be described as a chemical expression of the darkness because it is produced during the night in response to the lower light exposure [43,44].

Between 7 and 8 a.m., at the beginning of the day’s activity, the body is prepared to wake up due to a cortisol peak. This hormone prepares the body for the increase in energetic demands induced by activity [45]. Cortisol production is linked to the diminution of melatonin secretion, which normally inhibits cortisol secretion. Thus, the cortisol secretion cycle is the opposite of the melatonin one [46]. In the morning, another hormone, ghrelin, is secreted following a pulsatile rhythm. It has three peaks of secretion, approximately at 8 a.m., 1 p.m., and 6 p.m. [47,48]. Ghrelin regulates energy homeostasis by increasing appetite and food intake.

At 10 a.m., adiponectin begins secretion and ends at 8 p.m., with a peak secretion level attained at 11 a.m. Adiponectin is an important regulator of glucose and lipid metabolism. It improves glycolysis and fatty acid oxidation via the activation of AMPK, a kinase involved in the support of energetic homeostasis, and which also reduces hepatic glucose production. These mechanisms increase glucose use and insulin sensitivity and prevent fat accumulation [45,49].

During the afternoon, from 2 to 6 p.m. and especially at the 4 to 5 p.m. peak, insulin is produced by pancreatic islets and induces metabolic changes: the catabolic reactions mediated by adiponectin become anabolic processes [50]. Insulin stimulates substrate storage by activating fatty acid synthesis genes (such as acetyl-CoA carboxylase) and glycogenesis genes, and by inhibiting gluconeogenesis gene transcription and fatty acid oxidation [9].

Furthermore, leptin peaks at approximately 7 p.m., after rising 3 h earlier (4 p.m.), and later declines back to baseline levels at 2 a.m. It removes food intake, increases lipolysis, and inhibits fat accumulation [45].

Finally, melatonin is once again produced at 10 p.m., marking the beginning of the resting phase. During this stage at 4 a.m., another hormone, the fibroblast growth factor (FGF)-21 is secreted by the liver. It peaks at 6 a.m. and declines back to nadir at 9 a.m. FGF21 is a key regulator of energetic homeostasis, it promotes AMPK activation, leading to fatty oxidation, glycolysis increasing, and substrates stockage inhibition [51,52,53]. All of these hormones regulate an alternating between catabolic and anabolic stages, essential to performing physiological processes at the optimal time of the day. For example, digestion and metabolization of nutrients are more efficient when the food is consumed earlier in the day during the active phase rather than during the resting phase [54,55].

If we refer to the 24-h hormone oscillations, we hypothesize that food intake should begin at 8 a.m., after the cortisol peak when the activity phase started, and should end no later than 6 p.m., during the ghrelin and insulin peak. Indeed, between 8 a.m. and 4 p.m., FGF21 and adiponectin are produced and promote fatty acid oxidation, glycolysis, and inhibit fat accumulation [53,56]. The consumption of food should not occur during the insulin peak because it induces fat storage, nor should it occur at night when leptin is produced, since it normally induces satiety [9,57]. Several hormone levels peak during the activity phase, suggesting early daytime is more optimal for food intake than evening [58]. Thereby, the optimal time for food intake seems to be during the morning and the early afternoon. This proposal needs to be confirmed by additional scientific studies carried out with animal and human protocols.

Melatonin secretion (and consequently cortisol production) depends on the photoperiods which are modified according to seasonal variations. At the European latitude, the duration of melatonin secretion is longer in winter, which causes the resting phase to extend, and is shorter in summer when the duration of the activity phase increases [59]. In theory, food intake should be adapted to seasonal variations to match the change in the duration of the activity period. A reduction in the feeding time should occur during the winter to correspond with the decrease in duration of the activity phase; the inverse is true during the summer. This phenomenon is observed in mammals inhabiting temperate latitudes where seasonal variations occur. They present a winter phenotype with a food intake reduction during short photoperiods and a summer phenotype with increased food consumption during long photoperiods [60]. However, an abundance of evidence has shown that the Homo sapiens is also a seasonal species, with an annual pattern of susceptibility to illness or mood changes, so it appears relevant to suppose that humans have to adapt their food intake to the seasonal variations [61,62,63,64].

Globally, metabolic hormones, circulating nutrients, and visceral neural inputs transmit rhythmic cues that permit brain and peripheral organs to be synchronized to feeding time. However these chrono-disruptions, mistimed eating, coupled with food abundance and electrical lighting have clearly deleterious effects on metabolic health [65]. Indeed, everyone has their own habits and is able to change their circadian rhythm by adopting an abnormal eating routine, bad sleep pattern, night shift, or jet lag [55]. This circadian misalignment can often increase the risk of developing obesity, diabetes, and cardiovascular disease [34,66,67].

## 5. Modern Lifestyle and Circadian Disruption: Leaving the Door Open to Metabolic Diseases

Modernization of human life refers to the transition from a “traditional” society to a “modern” society. It is deeply linked to industrialization and urbanization processes. It began in the 19th century when the First Industrial Revolution hallmarked a boom mechanical invention, allowing for production optimization and cost reduction. The invention of the steam machine gave birth to factory development that rapidly invaded the countryside, transforming the rural environment into an urban one [68]. Other industrial revolutions came after the first one and lead to considerable changes to human life. Electrical lighting was invented by Thomas Edison in 1879; vehicles such as trains, cars, and boats were modernized; agriculture became mechanized and the invention of fertilizers increased farming production after the 2nd World War. At the same time, the food industry experienced a boom in the 1970s with the arrival of large retailers, a success that was further accelerated by the mass shift to household fridges and the expansion of the frozen food market [69].

Finally, all these revolutions led to a new age of globalization in the 21st century, characterized by a rapid growth of the global economy, increasingly faster means of transport, and the emergence of new technologies such as satellites for telecommunication, television, computers, internet, or smartphones [70].

Although these industrial revolutions have driven progress in many sectors, they have also caused substantial changes to the human routine, especially human circadian rhythms.

Firstly, the invention of the electric light had major consequences on the way people worked and lived because they were now able to work at night. Globalization and technological advances also promote night shift because many companies relocate to countries situated in different time zones, forcing company employees to synchronize their tasks with the work schedule of the countries they operate for [71]. Almost 30% of adult employees work outside daytime hours (between 9 a.m. and 5 p.m.) and 19% of European workers reportedly work at least 2 h between 10 p.m. and 5 a.m. [72].

Being awake at night leads to circadian disruption by modifying hormone levels. Several studies have focused on the hormone variations experienced by night workers, and have provided evidence of melatonin and cortisol changes: melatonin levels are lower in the night worker groups [73,74], and the total levels of cortisol production are reduced in shift workers [75,76].

There is evidence that circadian rhythm disturbances induced by changes in night-time and day-time patterns are a risk factor for cardiovascular diseases. Shift work is associated with a higher risk of coronary heart diseases and vascular events such as myocardial infarction or ischemic stroke, in comparison to day work [77,78].

These observations have also been noted in animal models: circadian rhythm disorganization induces cardiomyopathy in hamsters and cardiac dysfunction in mice [79,80]. Artificial lighting has also changed human lifestyles by interfering considerably with individual sleep preferences among the population. Most people accumulate a lack of sleep during the work/school days and compensate by extending sleep duration on weekends. This mismatch between weekday and weekend times of sleep duration leads to a circadian clock disordering known as “Social Jetlag”, where the endogenous circadian clock does not match with actual sleep times [81]. This phenomenon is associated with cardiovascular risk factors, with higher triglyceride and fasting insulin levels and lower high-density lipoprotein (HDL) cholesterol. These components predispose one to the development of obesity, diabetes mellitus, and atherosclerotic cardiovascular disease [82,83,84].

Furthermore, changes and progress induced by industrial revolutions have modified the food habits of people by impacting their timing of eating, the number of meals per day, and the qualitative aspect of the consumed food [85]. Artificial lighting, night shift, social jetlag, and later bedtimes tend to postpone mealtimes (especially the evening dinner), induce meal omission (usually breakfast), or increase snacking between regular meals [86,87].

The time at which one eats is really important for metabolic health because late-night eating (defined as eating dinner within 2 h of bedtime) is associated with increased body fat, leading to a high risk of obesity [88,89]. Moreover, the authors observed that people with late dinner habits are more susceptible to consume larger portion sizes, second rounds, and energy-rich foods; these people also present a high fat mass, insulin resistance, and cardiovascular risks [90,91]. In animal studies, authors showed that food availability during the day (where rodents are normally supposed to sleep) induced circadian clock disruption and thus metabolic disorders [92,93,94].

Late-night eating is also a risk factor for insulin resistance development. Glucose tolerance, for an identical meal, is higher in the morning (8 a.m.) than in the evening (8 p.m.), and similar rhythms have been observed in rodent models [95,96]. During the resting phase, insulin levels are reduced to the offset and beta-cell responsivity to glucose is lower. If glucose consumption occurs during the evening, the body will not be able to process it properly, leading to lower insulin sensitivity [96,97].

In addition, processed and ultra-processed foods enriched in fats, salt, and sugar, are positively associated with being overweight and obese [98]. Foods with added sugar, sweeteners, and/or saturated fats raise blood levels of low-density lipoprotein (LDL), glucose, and insulin, and these level profiles are associated with an increased risk of coronary heart diseases, glucose intolerance, and insulin resistance [99,100]. The consumption of these types of food associated with late-night dinner could be an aggravating risk factor of obesity, CVD, and diabetes [101].

In the same way that eating at an inappropriate time promotes metabolic risks, missing meals normally consumed during the activity phase can have consequences on the health. Numerous studies highlight an association between skipping breakfast and weight gain, cardiovascular risks, and diabetes, supporting the fact that breakfast consumption is essential to a healthy eating regimen [102]. Breakfast skippers are associated with a higher body mass indices and higher levels of hemoglobin A1C (HbA1C), another marker for glucose metabolism dysfunction), leading to an increased risk of obesity and diabetes [103,104]. They also present elevated blood pressure and increased levels of LDL which indicate a higher risk for CVD [105,106].

In light of these elements, it is clear that circadian clock disruption, caused by changes in work schedules and food habits, has significant effects on metabolic health. A solution to reduce the risks of developing metabolic disease could be the implementation of intermittent fasting to realign lifestyle behaviors with the biological circadian rhythms.

## 6. Association between Time-Restricted Feeding and Normal Circadian Rhythms: A Relevant Approach for the Fight against Metabolic Diseases

The hypothesis regarding the effectiveness of time-restricted feeding in metabolic disorders is that imposing eating/fasting cycles will restore robust circadian rhythms and improve metabolic homeostasis. Several clinical studies as well as studies with animal models were carried out to investigate metabolic disorders [14,15,27,107,108,109,110,111,112,113,114,115,116,117]. Some reviews evaluated the metabolic effects of time-restricted feeding in both animal and human studies; without focusing exclusively on the populations with metabolic syndrome [24,118,119,120].

Regarding the animal models, it has been shown in rodents that time-restricted feeding during the animal activity phase, significantly protects the mice from diet-induced obesity and associated metabolic complications, while also improving glucose tolerance and reducing liver weight [121]. Indeed, it induced a significant decrease in body weight, associated with a loss of fat mass in C57BL6/J mice [122,123,124,125]. The reduction of fat mass could be explained by an increased AMP-activated protein kinase (AMPK) activity induced by fasting, because its kinases promote fatty acid oxidation and inhibit acetyl CoA carboxylase (ACC), one of the enzymes involved in fat storage [122]. The mice also experienced decreased levels of insulin and fasting glucose levels, suggesting an improved glucose metabolism and insulin sensitivity [122,123,124,125,126]. Time-restricted feeding revealed beneficial effects in rat models as well [127,128]. The authors observed that it induced weight loss due to a significant reduction of visceral and subcutaneous adipose tissues and a better activation of PGC1α, a transcriptional coactivator involved in mitochondrial biogenesis and fatty acid oxidation. Rats improved glucose tolerance and had lower LDL levels and higher HDL levels, showing an improvement in CVD risk markers [127,128].

Beneficial results regarding obesity were also observed in human studies when this nutritional strategy was applied during the activity phase. This strategy could be divided into early and late time-restricted feedings with different results. Indeed, the early time-restricted feeding, where the food intake occurs between 8 a.m. and 6 p.m., facilitated weight loss and appetite reduction in overweight and obese people [14,111,114,115]. Prediabetic and diabetic patients, characterized by an impaired glucose tolerance, presented lower insulin levels and a better insulin sensitivity when they restrained their daily eating window to 8 h per day [14,15,115]. Moreover, it induced changes in cardiovascular markers by decreasing blood pressure and LDL levels [14,111]. The changes observed among the participants of these studies are beneficial for their metabolic health. A decrease in bodyweight and loss of fat mass are associated with an improvement in health-related quality of life, reduction in obesity risks, and comorbidity development [129]. Moreover, it is well known that a decrease in blood pressure and LDL levels is associated with better cardiovascular health, whereas a higher insulin sensitivity reduction decreases the risk of insulin resistance and diabetes [130]. The global mechanism behind the beneficial effects of early time-restricted feeding involved the central and peripheral circadian oscillators. Indeed, when food is consumed between 8 a.m. and 6 p.m., the secretion of hormones from the peripheral oscillators are in phase with the central mechanism of synchronization emerging from the suprachiasmatic nucleus [131,132,133]. These mechanisms should optimize the functioning of the peripheral organs involved in the metabolism regulation thus preventing the development of type 2 diabetes, as it can be observed when food intake is matched with metabolic hormones’ oscillations [14,15,115]. It will be an interesting challenge to identify the signal transduction pathways participating in the impulsion of peripheral clocks by the central clock in the case of early time-restricted feeding.

All of these elements highlight the importance of an alignment between food intake and circadian rhythms to improve cardiometabolic health. Indeed, several studies used late time-restricted feeding where subjects were allowed to eat until the evening and showed reduced beneficial effects. Results on weight loss are mixed, showing a weight loss [116] or no significant change in weight or whole-body fat mass [27,117]. They also showed no significant effects on fasting glucose, HbA1C, triglycerides, total cholesterol, blood pressure, LDL, or HDL levels [27,116,117]. These outcomes demonstrate how important matching the eating window and circadian rhythms is to optimize the effects of time-restricted feeding and, therefore, to provide health benefits.

However, it would be more relevant to compare the effects of early time-restricted feeding with those of late time-restricted feeding to give a better understanding of the importance of the daily eating window moment for the favorable outcome of this strategy. For the moment, only one study has compared early (8 a.m. to 5 p.m.) versus late (12 to 9 p.m.) time-restricted feeding on glucose tolerance [113]. Authors demonstrate that time-restricted feeding improves glycemic responses, regardless of meal timing (late or early), although the early time-restricted feeding group has presented a lower mean fasting glucose than the late group [113]. Even if these results tend to discredit the idea of the importance of an early food intake during a time-restricted feeding, this study presents several limits. Only men were included so these findings cannot be extended to women. The number of participants is small (only fifteen), and the early and late time-restricted feeding groups were tested during a short period of time (one week).

More randomized clinical trials with larger cohorts and longer durations of time-restricted feeding treatment will be required to conclude on the most efficient differences strategy, especially between early and late time-restricted feeding. Despite the fact that more and more authors investigate the metabolic effects of fasting, a significant limitation persists. In most of the studies, participants had an imposed fasting duration, but they were free to choose the time frame that best fit their eating habits [107,108,109,110,112]. Some of these studies showed beneficial effects of fasting with a significant weight loss, waist circumference and blood pressure reduction, and lower LDL level [107,108,109]. Others presented less positive results, where time-restricted fasting did not confer significant effects on glucose or insulin levels [107,110,112], blood pressure [112], or weight loss [110].

These mixed results are most likely caused by a lack of control on the timing of participants’ feeding windows, which are spread over the day. As we have described previously, the timing of food intake is essential to obtain beneficial metabolic effects, so it is difficult to draw a real conclusion on the outcomes of time-restricted feeding based on these studies which do not impose a precise feeding window on their subjects. The lack of protocol standardization is a major gap in time-restricted feeding research.

## 7. Warnings and Limits of the Association between Intermittent Fasting and Normal Circadian Rhythms

Despite its numerous health benefits, intermittent fasting has its disadvantages and limits, which constrain its application. One of the most restrictive points is the daily eating window of 10 to 6 h, because it can be hard to observe while maintaining a family, social, or work life. Indeed, night shift work makes intermittent fasting difficult to respect due to desynchronized work schedules that disturb the everyday life routine [72]. Family life can also be a brake for time-restricted feeding because family mealtimes usually adhere to the common eating patterns of modern society (breakfast, lunch, and dinner). Dinner is actually the most important meal in family environments. It facilitates communication between family members and favors socio-emotional development and mental health [134]. Eating is a social activity and promotes socialization by meeting friends, colleagues, or relatives. Commensality, defined by the act of eating with others, provides opportunities for social integration, social support, and companionship [135]. Therefore, following a different feeding pattern than that of one’s entourage can be a hurdle to social and family development and can drive isolation, loneliness, or depression [136,137].

Another limit of intermittent fasting is the lack of protocol standardization. There is no consensus about the ideal timing for eating/fasting pattern or the optimal duration of each window. Studies use different durations for the feeding period, from 8 to 10 h, and recommend diverse time slots for time-restricted feeding [14,109,111,114]. Although there is no evidence of serious adverse effects of intermittent fasting when it is aligned with the circadian clock [109], several authors did observe the importance of timing when choosing the eating window. Indeed, time-restricted feeding increases metabolic health only when the eating window is matched with earlier hours. Intermittent fasting that situates the eating window during the evening is associated with a significant increase in bodyweight, fat mass, and glycemic levels. These values are associated with a higher risk of obesity and insulin resistance [138,139]. Unfortunately, research on time-restricted feeding is limited, and clear conclusions cannot be made at present.

Moreover, seasonal changes are not taken into consideration in the selection of the eating window whereas the activity phase is dependent on it. Eating hours should be adapted to the season and the length of days to match hormone oscillations and optimize physiological processes [60,140,141].

In spite of its positive results, the application of intermittent fasting needs to be rigorously supervised. Even if the timing of the feeding window is hard to respect because it interferes with social and family lives, it is essential to follow an early eating pattern. A desynchronization of time-restricted eating and the circadian clock with the evening meal window can reverse the beneficial effects of fasting.

## 8. Conclusions

**Figure 2:**
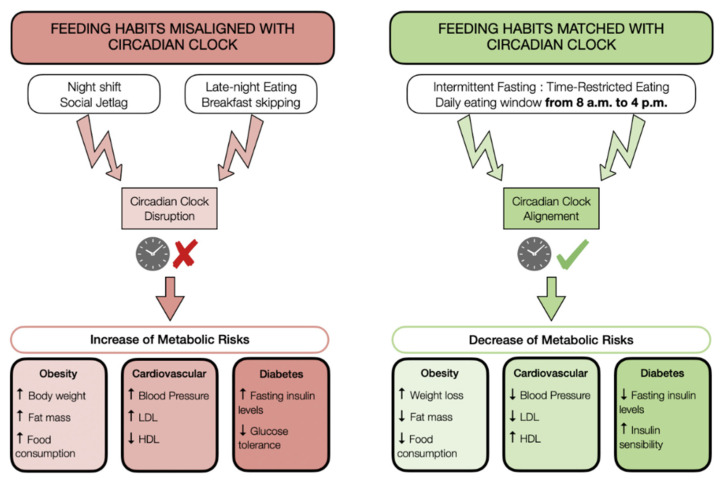
Impacts of food habits on metabolism are connected with the circadian clock. The way that people eat is essential for metabolic health. When food habits (timing and number of meals per day) are misaligned with the circadian clock, they prevent an increased risk of obesity, cardiovascular disorders, and diabetes. On the other hand, practicing time-restricted fasting where the eating window is aligned with circadian rhythms improves metabolic health.

Circadian rhythms play an essential role in regulating physiological processes (Figure 2). It accurately regulates several hormone levels 24 h a day, inducing a balance between catabolic and anabolic processes that are crucial to perform physiological activity at the optimal time of day [32]. However, modern habits, especially food habits such as skipping breakfast or late-night eating, are involved in circadian clock disorders that have major impacts on metabolic health. Indeed, circadian disruption leads to an increase in metabolic complications, such as obesity, CVD, or insulin resistance [101,102].

The time-restricted feeding approach appears as a relevant solution to restore metabolic health only when the feeding pattern is aligned with the circadian clock, that is during the activity phase. Early time-restricted eating, based on a limited feeding window per day, reduces cardiometabolic risks by inducing weight loss, and by decreasing markers of cardiovascular and diabetes risks [14,109,111,114].

The assembly of the presented results suggests that time-restricted feeding could be an alternative non-pharmacological strategy that could prevent obesity and its associated metabolic disorders. As research on time-restricted feeding is limited, future studies should be made in order to clearly established the optimal time and duration of the eating window in order to propose safe and efficient time-restricted feeding protocols.
